# Cell-free DNA as a complementary diagnostic tool for neglected tropical diseases towards achieving the WHO NTDs elimination by 2030

**DOI:** 10.1016/j.jlb.2024.100283

**Published:** 2024-12-13

**Authors:** Priscilla Osei-Poku, Lucienne Tritten, Fatima Fordjour, Alexander Kwarteng

**Affiliations:** a Department of Biochemistry and Biotechnology, Kwame Nkrumah University of Science and Technology (KNUST), Kumasi, Ghana; b Kumasi Centre for Collaborative Research in Tropical Medicine (KCCR), Kumasi, Ghana; c Institute of Parasitology, McGill University, Sainte-Anne-de-Bellevue, Quebec, Canada; d Department of Microbiology, University for Development Studies, Ghana

**Keywords:** Neglected tropical diseases, NTDs, Cell-free DNA, cfDNA, Global burden, Sub-Saharan Africa

## Abstract

Neglected Tropical Diseases (NTDs) continue to ravage the poorest regions of the world, with over 600 million people being affected in Sub-Saharan Africa. The global burden of NTDs within these regions is staggering, particularly post-COVID-19 pandemic, where the emerging infection intercepted the existing eradication efforts and protocols such as the Mass Drug Administration (MDA). This further complicated the approaches laid down to achieve the endgame program of eliminating the neglect and transmission of NTDs. To compensate for the detriment of COVID-19's interruption, accurate and timely diagnoses play a vital role in attaining the objectives of the WHO's goal of NTD elimination by 2030. To this effect, alternative approaches in diagnostics are urgently needed, particularly with the inadequacy of current diagnostic strategies for NTDs. Cell-free DNA (cfDNA) has shown great promise in detecting NTDs. Several studies have demonstrated its potential for diagnosing diseases such as malaria, leishmaniasis, and schistosomiasis. However, the adoption of cfDNA in NTD research faces several challenges, including the cost of the procedure, standardization, and technical expertise. Proper capacity building and training can mitigate some of these challenges. However, despite these limitations, the affordability of cfDNA detection is improving due to increased awareness of the approach and researchers' integration considerations into current diagnostic routines. In conclusion, while there are challenges to adopting cfDNA in NTD research, it remains a promising alternative strategy to be considered in the fight against NTDs.

## 1 Background

Neglected tropical diseases (NTDs) are a diverse group of parasitic and bacterial infections that tend to become chronic and debilitating and affect some of the world's most marginalized communities. The World Health Organization (WHO) identifies 20 NTDs, which are responsible for significant morbidity and mortality, including lymphatic filariasis, schistosomiasis, onchocerciasis, soil-transmitted helminthiases, and trachoma, among others [1]; [2]; [3]. They affect more than one billion people worldwide, particularly in low-income countries. NTDs are highly prevalent in sub-Saharan Africa, Asia, and Latin America [4], with Africa experiencing over 50 % of the world's NTD burden. For example, a parasitic infection caused by *Schistosoma* worms affects over 250 million people globally, with over 90 % of the cases occurring in sub-Saharan Africa [1]; [2]; [3].

Similarly, onchocerciasis, also known as river blindness, is endemic in 31 African countries, with over 20 million people infected. Other NTDs, such as lymphatic filariasis, trachoma, and soil-transmitted helminthiases, are highly prevalent in sub-Saharan Africa [1]; [2]; [3]. The associated complications and disability of these NTDs remain a significant public health challenge. They have far-reaching consequences, leading to substantial economic losses due to reduced productivity and healthcare costs. NTDs disproportionately affect the most impoverished populations, making it difficult for affected communities to break free from the cycle of poverty.

Additionally, the COVID-19 pandemic has had significant negative implications on NTD control programs. The pandemic intercepted mass drug administration (MDA) programs, community outreach services, and diagnosis services, thereby impeding the progress of NTD elimination targets. These disruptions have led to an increase in NTD cases, further highlighting the need for improved and sustainable diagnostic strategies since early diagnosis and treatment are crucial in controlling and eliminating these diseases [1]; [2]; [3]. With the goal of reducing morbidity and mortality incurred by NTDs, the WHO produced an initial roadmap in 2012 [5] aimed at controlling, eliminating, or eradicating 17 NTDs by 2020. The revised NTD roadmap 2021–2030 ([1]; [2]; [3]; *Ending NTDs Together towards 2030*, 2021), developed within the United Nations Sustainable Development Goals framework, aims to control and eliminate 20 NTDs by 2030. Currently, the control of many NTDs heavily relies on therapeutics, such as MDA. However, it is crucial to consider the most effective approach to reliably identify and reduce or eliminate the transmission of these infections.

A critical element of the management of infection and disease is the use of accurate diagnostic methods for the surveillance of infection and the potential co-infection with other pathogens to properly evaluate treatment efficacy or detect the emergence of drug resistance [6,7]. The current diagnostic strategies for NTDs, each with its advantages, are often expensive, invasive, and have limited sensitivity and specificity, making it a challenge to achieve the WHO's goal of NTD elimination by 2030, particularly with one of the pillars being to intensify cross-cutting approaches. Therefore, further research is needed to optimize and ensure the efficiency, accessibility, and affordability of molecular diagnostic tools in resource-limited settings. New molecular tools are urgently required to improve the diagnosis and monitoring of NTDs.

Liquid biopsy, utilizing cfDNA from bodily fluids, offers a non-invasive, real-time approach to monitor disease progression and treatment response. This makes it a valuable tool within the NTD framework, as it can support integrated disease management strategies. By enabling the simultaneous detection of multiple pathogens, liquid biopsy optimizes resource use and enhances surveillance capabilities. This method holds significant potential for detecting various NTD-associated pathogens, including *Schistosoma* spp. and *Leishmania* spp. Early diagnosis, facilitated by liquid biopsy, can significantly impact morbidity and mortality rates in endemic regions. While challenges such as technical variability, resource constraints, and regulatory and community acceptance issues exist, successful integration of liquid biopsy into health systems can significantly contribute to the control and elimination of NTDs. Hence, a novel molecular approach utilizing the detection of parasitic cell-free DNA (cfDNA) presents a promising diagnostic tool, offering the potential to overcome many limitations of existing diagnostic methods. Although the use of cfDNA in NTDs is still in its early stages, several studies have reported promising results. Certain challenges must be addressed before cfDNA detection can be widely implemented, especially in developing countries. This review evaluates current diagnostic approaches and emphasizes the potential integration of parasitic cfDNA as a standard diagnostic approach for various NTDs.

## 2 Current NTD diagnostic strategies and their limitations

The customary approach to screening parasites entails the observation of adult parasites using parasitological techniques. This involves clinical examination, microscopy, serology, and molecular methods such as polymerase chain reaction (PCR) and loop-mediated isothermal amplification (LAMP). These involve the detection of the parasite(s) in clinical samples such as blood, urine, and stool.

Microscopy remains the gold standard for many NTDs, such as malaria, helminth infections, and leishmaniasis [8,9]. Identifying parasite stages based on their morphology requires the expertise of highly specialized researchers. It may involve invasive tissue sampling from patients and microscopic examination, leading to time-consuming and labor-intensive procedures that can be hard to implement, especially in resource-limited settings [10].

Serological methods involve the detection of antibodies produced in response to infection. These methods include enzyme-linked immunosorbent assays (ELISAs), immunochromatographic tests (ICTs), and rapid diagnostic tests (RDTs). Although these serological tests mostly offer alternative diagnostics means, they are now routinely used by control programs [8]. These tests are easy to perform, require minimal training and equipment, and can be used to detect asymptomatic infections. However, they are limited by their inability to differentiate between past and current infections and the possibility of cross-reactivity with other diseases [10]. These methods present further limits, including low sensitivity and specificity, high cost, and technical expertise requirements. In some cases, they may also require the collection of invasive or large sample volumes.

Molecular methods involve the detection of parasite DNA or RNA in clinical samples using polymerase chain reaction (PCR) or loop-mediated isothermal amplification (LAMP) techniques. Historically, molecular techniques were primarily confined to research settings. However, recent advancements have ignited optimism about the potential for molecular assays to supplant traditional methods like microscopy and immunodiagnostics in detecting certain NTDs [9]. This paradigm shift could revolutionize disease diagnosis and management. These methods are highly sensitive and specific, can detect low levels of parasite nucleic acids, and offer multiplex capabilities (i.e., the simultaneous detection of multiple pathogens in a single assay). The various forms of PCR assays are highly adaptable. They only allow the detection of current ongoing infections and can easily be implemented to monitor the treatment efficacy of drug resistance issues [7]. The array of available chemistries may target different genetic markers, where the presence of each of them produces different fluorescent signals or produces a color change visible to the naked eye, as in LAMP assays. Despite the specialized equipment and expertise required, some PCR procedures can become true point-of-care tests [10]; [11]. Ideally, techniques such as PCR rely on specific primers, restricting their ability to detect only targeted species and making them incapable of identifying unanticipated parasitic species.

The limitations of these conventional techniques frustrate the elimination of NTDs within highly endemic regions that are, more often than not, regions of limited resources. Thus, the demand for more efficient and sensitive methods of screening parasites is increasingly critical. Amenable to high-throughput, liquid biopsies have recently become popular for diagnostic purposes.

Liquid biopsies represent a transformative platform for cancer diagnosis and management. By analyzing circulating tumor DNA (ctDNA), cell-free DNA (cfDNA), extracellular vesicles, and circulating RNA (cfRNA) from various bodily fluids such as blood, urine, and cerebrospinal fluid (CSF), these non-invasive tests offer significant advantages over traditional tissue biopsies [12,13]. With their potential for early detection, real-time monitoring of treatment response, and identification of actionable genetic alterations, liquid biopsies are revolutionizing personalized medicine [13]. This innovative technology enables clinicians to make more informed treatment decisions while sparing patients the burden of invasive procedures. They are now extensively used for the identification and monitoring of various types of cancer, such as lung, breast, colorectal, and prostate cancer [[14], [15], [16]]. In a study by Abbosh et al., the use of liquid biopsy for the detection of circulating tumor DNA (ctDNA) in plasma samples was shown to have a sensitivity of 90 % and specificity of 100 % for non-small cell lung cancer (NSCLC) [17]. Liquid biopsy has also been investigated for the early detection of cancer, such as early-stage colorectal cancer, with a sensitivity of 71 % and specificity of 98 % [18]. Among the molecules observed in liquid biopsies, cell-free DNA (cfDNA) mirrors molecular profiles similar to tumor tissue DNA [13,19], gaining increasing attention as a molecular target for detecting and monitoring other conditions, including NTDs.

## 3 Cell-free DNA

cfDNA refers to the fragmented nucleic acids released from cells into the extracellular space in various bodily fluids, such as blood and urine [20]. The discovery of cfDNA in human plasma dates back to the 1940s when Mandel and Métais first reported the presence of cfDNA in blood [21]. Circulating cfDNA in plasma and urine may serve as a biomarker of parasite burden. The presence of cfDNA in bodily fluids indicates parasite attrition or turnover, during which parasitic DNA is released into the bloodstream. A portion of this released DNA undergoes degradation and is subsequently excreted via the urinary system.

Numerous studies have been conducted to investigate the characteristics and potential applications of cfDNA in various disease contexts, including cancer [22], autoimmune diseases [23], and infectious diseases such as tuberculosis [24,25]. For example, cfDNA has been used in cancer diagnosis to detect genetic mutations and tumor-specific biomarkers. A study conducted by Liao et al. [26] demonstrated the use of cfDNA to detect tumor-specific mutations in patients with advanced cancer. The study showed that cfDNA analysis was more sensitive than traditional tissue biopsies for detecting mutations and guiding treatment decisions. A study conducted by Yu et al. [25] evaluated the use of cfDNA to diagnose tuberculosis (TB) in urine samples. The study showed that cfDNA detection had a sensitivity of 83.3 % and a specificity of 100 %, which was higher than the sensitivity of traditional TB diagnostic methods. WATSON, a CRISPR/Cas-13-based platform, accurately detected *Mycobacterium tuberculosis* cfDNA in 90 % of TB samples. As a liquid biopsy approach, it offers less invasive sample collection [27]. Currently, cfDNA has been integrated into the diagnostic approaches for various diseases. With the detection of cfDNA being recognized as a routine biomarker in oncology and prenatal diagnosis, several studies have explored the further use of cfDNA as a diagnostic marker for human parasitic infections [[28], [29], [30]]. The cfDNA of some parasitic pathogens has been reported in the serum of infected patients using conventional PCR-based methods [20,[31], [32], [33], [34]].

The discovery of cfDNA has opened new opportunities to explore non-invasive and specific diagnostic tests and their feasibility and affordability in resource-limited settings. Utilizing non-invasive clinical specimens such as urine and saliva offers a promising alternative to traditional invasive methods, promising to significantly enhance diagnostic accuracy, patient comfort, and accessibility to testing. However, these are yet to be fully proven. In this context, we provide an overview of the progress of the potential use of cfDNA as a diagnostic method in selected NTDs. Here, we describe the stand of research and needs for selected pathogens and the NTDs they cause.

## 4 cfDNA use in infections of medical relevance in tropical regions

### 4.1 Chagas disease

Chagas is a parasitic disease caused by *Trypanosoma cruzi* and transmitted by triatomine bugs. The diagnosis of Chagas disease is challenging due to the low parasitic burden and the chronic nature of the disease. Detecting *T. cruzi* DNA by PCR has shown promising results in diagnosing the infection. A study by Lozano et al. evaluated cfDNA as a diagnostic tool for Chagas disease in Argentina. The study profiled that the diagnosis of Chagas in chronically ill patients is limited by serological testing, which detects previous exposure rather than active infection. Thus, they evaluated the use of nucleic acids from serum extracellular vesicles and cfDNA to detect the active presence of the parasite in 448 serum samples from Chagas patients. They found that both approaches could be used to demonstrate the active presence of the parasite [35]. Another study by Duffy et al. [36] in Bolivia employed cfDNA detection as a diagnostic tool for Chagas disease. The authors suggested that cfDNA detection could be useful for diagnosing Chagas disease, especially in resource-limited settings.

### 4.2 Leishmaniasis

Leishmaniasis is a parasitic disease caused by *Leishmania* parasites and transmitted by sandflies. The diagnosis of leishmaniasis is often challenging. Diagnosis is made clinically with direct (parasitic) and indirect (immunologic) confirmation. However, advanced diagnostic testing, such as blood-based PCR, can enable earlier detection of *Leishmania* infections, even in asymptomatic cases. This could lead to earlier initiation of treatment, potentially improving patient outcomes and reducing parasite transmission [37]. Several studies have investigated using cfDNA as a diagnostic tool for leishmaniasis [28]. The high sensitivity of blood-based PCR allows for detecting low-parasitemia Leishmania infections, even in asymptomatic cases. This demonstrates the feasibility of Leishmania cfDNA detection [38]. Using cfDNA as a diagnostic tool for leishmaniasis has shown promising results in several studies. However, conventional PCR is still the gold standard for leishmaniasis diagnosis. cfDNA detection can be useful for leishmaniasis diagnosis, especially in situations where PCR is not available or not feasible. Further research is needed to standardize cfDNA detection methods and evaluate their diagnostic performance in different settings.

### 4.3 Malaria

Malaria is a parasitic disease caused by *Plasmodium* parasites and transmitted by *Anopheles* mosquitoes. The diagnosis of malaria is traditionally done by the microscopic examination of blood smears, which is time-consuming and requires trained personnel. Although only a few studies have explored cfDNA as a diagnostic tool for malaria, a study by Gal et al. [39] has validated the existence of cell-free *P. falciparum* DNA in plasma. A recent study demonstrated the feasibility of detecting *P*. *falciparum* infection through PCR analysis of human urine and saliva samples [40]. While this approach necessitates further refinement, it presents a promising avenue for implementing large-scale screening programs that minimize the need for invasive procedures such as blood draws.

Furthermore, another study by Nwankanma et al. [41] demonstrated the presence of parasite DNA in the saliva, blood, and urine of infected individuals. Saliva-derived samples exhibited the greatest potential as a minimally invasive diagnostic tool, presenting a new direction for malaria infection investigation. Imwong et al. [42] study revealed that plasma cfDNA levels, measured by direct PCR, can differentiate uncomplicated malaria from severe disease in children and adults. This is affirmed by recent studies that also showed elevated levels of circulating cfDNA in the plasma associated with severe malaria, differentiating it from milder forms of the disease [43]. This finding underscores the potential of cfDNA as a diagnostic biomarker for malaria severity.

Additionally, quantifying plasma cfDNA is a simple assay useful in identifying children at risk for fatal outcomes, thereby having a promising potential as a point-of-care assay [43]. Loop-mediated isothermal amplification (LAMP) has been employed to detect *P*. *falciparum* and *P*. *vivax* in the urine and saliva of malaria patients. While this technique offers potential advantages, a comparison with nested PCR revealed a lower sensitivity of LAMP for detecting *Plasmodium* DNA in these non-invasive samples [44]. In a simpler sense, while cfDNA detection via LAMP can be a complementary diagnostic tool, particularly in resource-limited settings, conventional PCR remains the gold standard for definitive malaria diagnosis.

### 4.4 Schistosomiasis

Schistosomiasis is a parasitic disease caused by *Schistosoma* parasites and transmitted by freshwater snails. The diagnosis of schistosomiasis is challenging due to the low parasitic burden and the variable clinical presentation. The gold standard for schistosomiasis diagnosis remains the parasitological detection of *Schistosoma* spp., eggs in fecal samples, typically achieved through the Kato-Katz technique. While this method is reliable, its sensitivity limitations, particularly in cases of low-intensity infections, necessitate the development of more sensitive diagnostic tools. This extracellular schistosome DNA, cfDNA, identified in bodily fluids holds significant potential as a diagnostic marker for active schistosomiasis infections, particularly in cases of low parasite burden where traditional diagnostic methods may be limited [20,31]. In that regard, PCR has emerged as a promising alternative, demonstrating superior efficacy in detecting low-intensity infections [[45], [46], [47]]. In a study by Hussein et al. [31], cfDNA detection of *S. mansoni* showed significant sensitivity and specificity. The authors suggested that cfDNA detection could be useful for schistosomiasis diagnosis, especially in resource-limited settings.

A direct comparison of Kato-Katz and PCR methods revealed a significant advantage for PCR. Using an artificially prepared positive fecal sample with a low egg count, PCR successfully detected *S. mansoni* DNA, while the Kato-Katz method was negative, highlighting the superior diagnostic accuracy of PCR. Additionally, the amplification reaction demonstrated high specificity, with no cross-reactivity observed with DNA from other helminth species. These findings suggest that the developed PCR assay could serve as a valuable alternative diagnostic tool for *Schistosoma* spp. infections [48]. Remarkably, LAMP assays have enabled the detection of *S. mansoni* DNA in stool samples during the earliest stages of infection in experimentally infected mice. This represents a significant advancement, as early detection is unattainable using traditional parasitological or serological methods [49]. Similarly, digital polymerase chain reaction (ddPCR) allows the detection of *S. japonicum* in various samples, including stool, with high sensitivity but suboptimal specificity [50]. In cases of refractory imported schistosomiasis, cell-free schistosome DNA was detected in bodily fluids through conventional and sequence capture PCR. Notably, while parasite ova were rapidly cleared from the urine, the persistence of parasite DNA in multiple specimen types, including non-fecal samples, post-treatment was observed. These findings suggest the potential utility of schistosome cfDNA as a biomarker for monitoring treatment response and identifying cases of treatment failure [32]. cfDNA detection of *Schistosoma* spp. may become useful for schistosomiasis diagnosis, especially in resource-limited settings.

### 4.5 Filariasis

Filariasis is caused by filarial nematodes and transmitted by mosquitoes. The diagnosis of filariasis traditionally involves microscopic examination of blood samples for the presence of microfilariae. PCR to detect filarial DNA as a diagnostic tool has opened new opportunities for seeking more non-invasive diagnosis of filariasis. Nematode-derived cfDNA was found in patient blood, providing a sensitive and specific diagnostic target for filariasis [51]. The complex life cycle of the filarial parasites and the periodicity of microfilariae in the blood present a challenge to their diagnosis. However, PCR has shown promising results in the diagnosis of filariasis. Unlike traditional diagnostic methods reliant on detecting live parasites, cfDNA can be detected in circulation regardless of the parasite's lifecycle stage, making it a potential biomarker for continuous monitoring of infection status [52]. A study by Lucena et al. [52] reported the possibility of detecting *W. bancrofti* cfDNA in blood and urine during daytime via PCR, allowing the convenience of sample collection during the day as opposed to night, which coincides with the appearance of the microfilariae in the blood. A study conducted by Rao et al. [53] compared the diagnostic performance of conventional PCR with real-time PCR amplification of cfDNA in plasma samples from patients with lymphatic filariasis. The work showed comparable sensitivity in detecting filarial DNA, although the real-time PCR was higher in selectivity. A recent study demonstrated the feasibility of detecting *W*. *bancrofti* DNA in urine samples as a diagnostic tool for filariasis. By employing conventional and semi-nested PCR methodologies, researchers explored the potential of this non-invasive approach for identifying patients infected with this parasitic worm [33].

In *Onchocerca volvulus* infection, skin snip evaluation done by Macfarlane et al. [54] showed to be of low sensitivity, especially in individuals with low skin microfilaria densities, hence, suggested circulating parasite-derived nucleic acids as an alternative method. The study analyzed two parasite-derived miRNAs, cel-miR-71-5p and bma-lin-4, and O-150 repeat DNA and showed that *O. volvulus* nucleic acids are variably detectable at low to undetectable concentrations in host plasma, making these markers inappropriate alone as diagnostic and monitoring markers of onchocerciasis and treatment, respectively. Others showed that plasma samples yielded no detectable Ov16R; however, urine-derived cfDNA emerged as a more promising target for identifying *O. volvulus* infection [55].

Targeting the *Loa loa*-derived LL2634 DNA fragment proved efficient at detecting the infection in plasma-derived circulating cfDNA from 48 out of 53 infected, microfilariae-positive patients using qPCR [55]. The detection of cfDNA in urine was possible, but the infection was not detected systematically. The inability to detect LL2643 reliably in a microfilaremic but presumably infected individuals was hypothesized to be due to an insufficient contribution of cfDNA by the adult parasites or to the nature of the sample storage conditions. The poor sensitivity of urine-based detection of *L. loa* cfDNA may be related to the nature of highly fragmented cfDNA in urine (40–250 bp) [55]. Interestingly, LL2643 cfDNA became undetectable within one month following diethylcarbamazine treatment, and treated individuals remained negative for at least a year.

## 5 A role for cfDNA in NTD elimination programs in endemic regions?

NTDs disproportionately affect impoverished populations, with a significant overlap between NTD prevalence and extreme poverty [4]. While the WHO's NTD road map prioritizes diagnosis, monitoring, and the programs' evaluation [1]; [2]; [3] , investment in diagnostic development remains critically low, constituting only 5 % of NTD research and development expenditure, a figure that has declined by 10 % over the past decade. Traditional NTD diagnostics are hindered by the absence of a gold standard and suboptimal sensitivity and specificity. We propose that cfDNA analysis could improve disease diagnosis and control as an integral part of the End Game Program of NTDs in endemic countries. However, the feasibility of using cfDNA analysis as a routine diagnostic tool in resource-limited settings will depend on various factors, including the availability of resources, adaptability to point-of-care tests, capacity building, and local expertise. One of the advantages of cfDNA analysis is its ability to detect low parasitemia in patient samples, making it a highly sensitive diagnostic tool [28]. This could improve infection detection and control by identifying asymptomatic carriers for timely interventions toward preventing further transmission. Moreover, developing point-of-care cfDNA tests could reduce the turnaround time for test results and improve patient management.

## 6 What do we need to adopt cfDNA to support NTD management?

Adopting cfDNA as a diagnostic tool for NTDs can potentially improve disease diagnosis and management. However, cfDNA has limitations, especially its integration into current diagnostic approaches for NTDs. Whether cfDNA analysis performs sufficiently well for diagnosing tissue-dwelling pathogens (and in which sample type(s)) remains to be determined. In line with this, knowledge of the cfDNA half-life and turnover while in circulation would facilitate data interpretation. Finally, how these cfDNA molecules are transported and protected from nucleases would help refine the development of the method.

In a 2019 review article, Olatunbosun et al. discussed the challenges and prospects of cfDNA analysis for NTD detection in developing countries. The authors noted that despite the potential benefits of cfDNA analysis, its implementation in these countries faces several challenges that can hinder the adoption and implementation of this technology in these regions [56]. Thus, its feasibility should be addressed duly, particularly within resource-limited regions.

One challenge is the need for more general standardization of cfDNA detection methods (gold standards). Standardization will be necessary to ensure the reproducibility and reliability of cfDNA as a diagnostic tool. Another is the availability of expertise and the need for capacity building for cfDNA analysis in low-resource settings. Most NTD-endemic regions lack laboratory facilities and, comparatively, personnel with the expertise to perform cfDNA analysis. Therefore, to push for the incorporation of cfDNA detection as a routine diagnostic tool, efforts should be made to build the capacity of local researchers and clinicians to perform cfDNA analysis and interpret results.

Commercially available tests often require laboratory infrastructure, limiting the accessibility of even the existing diagnostics in resource-constrained settings (Hotez et al., 2016). cfDNA analysis by next-generation sequencing (NGS) offers excellent potential, but current commercial panels can be expensive due to high sequencing requirements. For example, Guardant360 has an estimated cost per sample of over US $1000 [57]. While NGS analysis of cfDNA can be costly, qPCR-based methods offer a more affordable and practical approach for wider applications, especially in resource-limited settings. Targeting cfDNA with qPCR can significantly reduce costs by over 100 times compared to NGS, making it a more feasible option for routine clinical use. To be valuable in the Endgame Program of NTDs, cfDNA analysis technology must be more affordable, less complex, and require minimal infrastructure in endemic countries. This will facilitate its implementation in resource-limited settings, mostly regions left behind by socioeconomic progress. The choice of a molecular method for diagnosis depends on factors such as available infrastructure and cost-effectiveness.

Notwithstanding the cost of cfDNA analysis, it remains relatively higher than traditional diagnostic methods while reducing labor investments. For instance, in a comprehensive cost analysis of molecular methods for diagnosing *Schistosoma* infection considering all relevant factors per sample, the single stool Kato-Katz method was the least expensive diagnostic approach ($6.89), followed by point-of-care circulating cathodic antigen (POC-CCA) ($7.26) and triplicate stool Kato-Katz ($17.54) [58]. Whereas the Kato-Katz technique in diagnosing *Schistosoma* infection is relatively less expensive, PCR-ELISA offers higher accuracy, and PCR-ELISA reagents cost approximately US$10 per fecal sample [59]. Therefore, finding alternative strategies for reducing the cost of reagents and equipment and promoting the use of shared resources and facilities through strong industrial-clinical collaborations can improve the affordability of cfDNA analysis. We suggest reinforcing research efforts to then integrate the technology into existing healthcare systems and encourage partnerships between governments, private companies, non-profit organizations, and educational programs. This will increase access and awareness of this technology in NTD endemic countries, thereby reducing costs due to increased demand for and production of its kit, including the reagents.

**Fig. 1:**
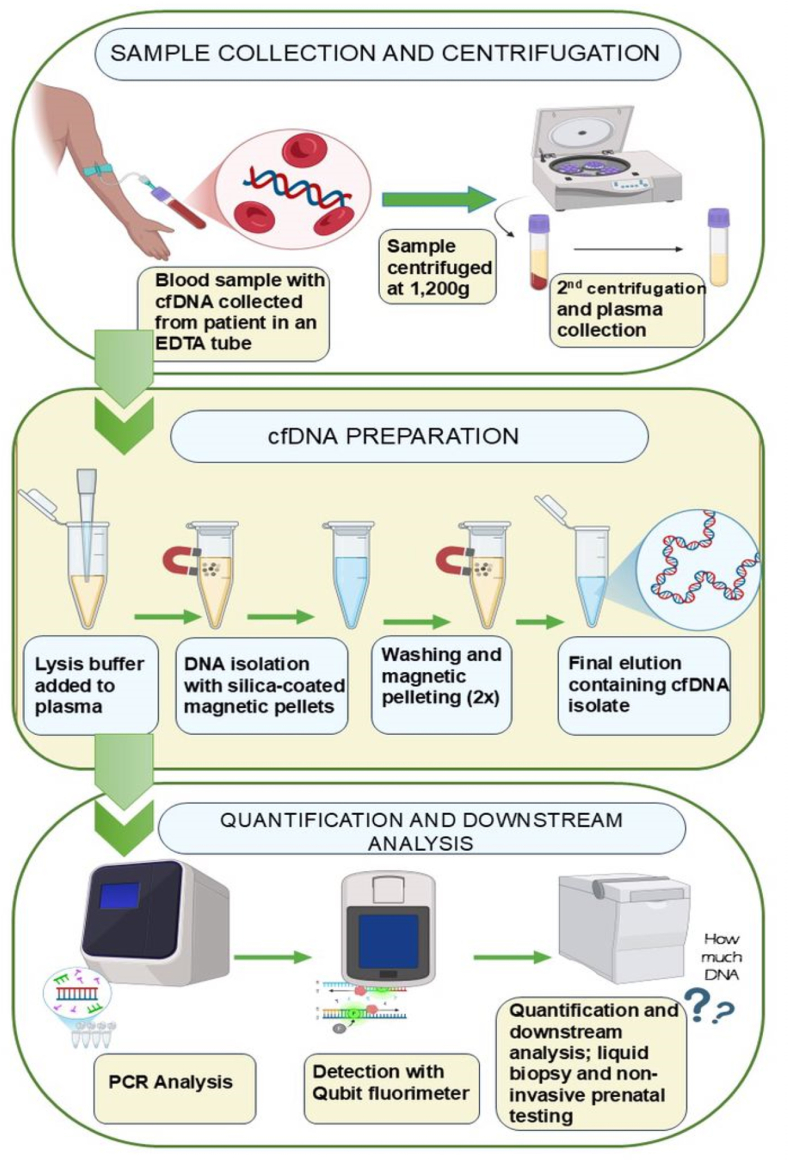
A flow chart representing cfDNA analysis.

Furthermore, while Kato-Katz offers a rapid turnaround time, typically within a few hours, the turnaround time for cfDNA analysis is another challenge since cfDNA analysis involves several steps (Fig. 1). cfDNA analysis using molecular methods often requires several days or even weeks for results due to the complexity of sample preparation and analysis. They vary depending on the specific method used, laboratory workload, and the availability of necessary equipment and reagents. The preanalytical step is critical for optimum translation to clinical practice, particularly in diagnosis. The sensitivity and specificity of cfDNA analysis can be affected by sample collection, storage, and processing; therefore, it may require additional knowledge, standardization, and expertise to obtain accurate results [60]. Proper attention and consideration must be given, especially regarding the choice of methods and equipment, the disease being diagnosed, the localization of the cfDNA-producing parasites stages (blood, tissues, intestine, etc.), the sample type, and the resources available.

By addressing these limitations, cfDNA would be the right approach to revolutionize the diagnosis of NTDs towards NTDs elimination. While, for instance, approaches such as Kato-Katz may remain the appropriate approach for broad systematic screenings, moving toward disease elimination usually involves moving from mass to targeted screening with assays that have better sensitivity and specificity [61,62]. Hence, cfDNA may be exactly the tool needed for the end-game stage. Thus, further studies are required to optimize the use of cfDNA in NTDs, ensuring its feasibility and effectiveness in NTD endemic regions and its efficiency in achieving NTD elimination.

## 7 Conclusion

The prospects of cfDNA analysis for NTD detection in developing countries are promising. The integration of cfDNA into current diagnostic approaches has gained attention in recent years. It can potentially improve disease diagnosis and management and hasten the achievement of the NTDs’ elimination goals. In this review, we discussed previous studies that have integrated the detection of cfDNA in diagnosing some parasitic infections and highlighted the associated challenges that ought to be addressed for efficient adoption as a routine diagnostic for NTDs. Despite the clinical benefits, the widespread adoption of parasitic cfDNA detection faces several challenges. These include high costs, lack of standardization, limited expertise, insufficient infrastructure, and long turnaround times, particularly in resource-limited settings where many neglected tropical diseases are endemic. It is essential to address these challenges in order to successfully integrate the detection of parasitic cfDNA into routine clinical practice. Strategies such as implementing cost-effective technologies, establishing training programs, and fostering partnerships between research institutions and healthcare providers can help overcome these barriers and improve access to advanced diagnostic tools for neglected tropical diseases. Further research is needed to assess cfDNA diagnostic performance in various settings, especially in low-resource areas, to promote its use.

## Ethical approval/patient consent confirmation

No ethical approvals or patient consent were necessary for the study.

## Declaration of competing interest

The authors declare that they have no known competing financial interests or personal relationships that could have appeared to influence the work reported in this paper.
